# A Multi-Center, Open-Label, Single-Arm Study to Investigate the Early Effectiveness of Esketamine Nasal Spray in Patients with Treatment-Resistant Depression Using a Mobile Self-Monitoring Application

**DOI:** 10.3390/ph17091143

**Published:** 2024-08-30

**Authors:** Junhyung Kim, Seung-Hoon Lee, Cheolmin Shin, Kyu-Man Han, Sung Joon Cho, Narei Hong, Changsu Han

**Affiliations:** 1 Department of Psychiatry, Kangbuk Samsung Hospital, School of Medicine, Sungkyunkwan University, Seoul 03181, Republic of Korea; jhcabilover@gmail.com (J.K.);; 2 Department of Psychiatry, Korea University Guro Hospital, College of Medicine, Korea University, Seoul 08308, Republic of Korea; 3 Department of Psychiatry, Korea University Ansan Hospital, Korea University College of Medicine, Ansan 15355, Republic of Korea; 4 Department of Psychiatry, Korea University Anam Hospital, Korea University College of Medicine, Seoul 02475, Republic of Korea; 5 Department of Psychiatry, Hallym University Sacred Heart Hospital, Anyang 14068, Republic of Korea; winghong@hanmail.net

**Keywords:** treatment-resistant depression, esketamine, mobile health, early effectiveness, self-monitoring

## Abstract

This study assesses the early effectiveness of esketamine nasal spray (ESK) in adults with treatment-resistant depression (TRD) 1 day after the first administration, as monitored through self-assessment via the mobile application, Esketamine Continuing Assessment for Relapse Prevention (EsCARe). In this multi-center, open-label, single-arm study, adults aged 18–65 years diagnosed with TRD after failing at least two antidepressant therapies were enrolled from five tertiary hospitals in South Korea. During the induction period, participants self-administered ESK twice weekly and used the EsCARe app daily to record mood, sleep, and somatic symptoms. Key clinical assessments, the Patient Health Questionnaire-9 (PHQ-9), the Hamilton Depression Rating Scale (HAMD), and the Generalized Anxiety Disorder Scale (GAD-7), were measured at baseline and at weeks 2 and 4. The reliability and validity of EsCARe was assessed. The treatment results indicated significant improvements in depressive and anxiety symptoms, with notable reductions in the PHQ-9 and the GAD-7 by week 2, and the HAMD by week 4. The EsCARe app reliably and validly monitored depressive symptoms and demonstrated a significant reduction in depressive symptoms 1 day after the first administration of ESK. Using ESK, complemented by mobile self-monitoring, effectively reduces the symptoms of TRD early in the treatment course. Integrating mobile health technology into the therapeutic regimen highlights a significant advancement in managing TRD, offering patients and clinicians immediate feedback on treatment efficacy.

## 1. Introduction

Major depressive disorder (MDD) is a leading cause of global disabilities, with the largest total number of years of life lost and an association with premature mortality. Thirty percent of patients with MDD do not respond to various antidepressants, nor do they reach remission, and are thus classified as having treatment-resistant depression (TRD) [1]. Patients with TRD experience a variety of treatment changes throughout their clinical course. However, the effect of antidepressants usually only appears after a few weeks. During this time, patients often experience symptoms of depression and are exposed to the risk of suicidal behaviors or self-harm [2]. Therefore, it is necessary to develop a treatment that will quickly relieve the symptoms of depression.

Esketamine nasal spray (ESK) is an FDA-approved drug for TRD in adults. The rapid effects of ESK in patients with TRD and suicidal ideation have been consistently reported in several randomized controlled trials [3,4,5]. ESK plus newly initiated oral antidepressants resulted in a rapid and time-increasing response as measured by the Montgomery–Åsberg Depression Rating Scale (MADRS), compared with placebo plus newly initiated oral antidepressants [6]. The interventional study, which used both MARDS and self-reported symptom assessments, such as the Patient Health Questionnaire-9 (PHQ-9), demonstrated the efficacy of ESK in patients with TRD [7]. Real-world data studies have consistently validated ESK’s effectiveness using self-reported symptom assessments [8,9]. ESK (co-administered with an antidepressant) is considered efficacious in the management of TRD by a World Psychiatry Association expert consensus [10]. The research indicates that esketamine could be a valuable medication to address the effectiveness gap caused by the slow beginning in the action of traditional antidepressants.

Although the MADRS is a validated measure and a brief and efficient tool, it takes 20–30 min to complete, making it difficult to use at both baseline and on day 1 in an outpatient setting. In addition, the fact that it is measured directly by the rater, who knows less about what the patient is experiencing, makes it challenging to use for a day 1 improvement assessment in the real world. This gap underscores the need for tools to capture the daily fluctuations in symptoms, particularly in a real-world setting outside of the clinical environment. In real-world clinical settings, it may be difficult for patients to attend a clinic visit the day after administration or for evaluators to perform assessments immediately [11,12]. Therefore, it would be helpful in many real-world clinical settings to identify a rapid response to esketamine through a self-reported tool that can assess daily mood swings without needing a clinic visit.

A methodological agreement consensus for evaluating an early therapeutic response in MDD has not been reached since rapid treatment response is not a significant concern in the clinical studies of existing antidepressants. Clinical assessments typically rely on retrospective self-reports, where patients recount their symptoms and emotional experiences from the preceding weeks. However, recent research indicates that these tools may not capture the critical details about symptom dynamics, including mood variations and symptom intensity fluctuations over time [13,14]. The Ecological Momentary Assessment (EMA) is a research tool that attempts to fill this gap by obtaining daily information about an individual’s emotional states, activities, surroundings, and natural environments [15,16]. EMA reduces recall bias and more accurately reflects the experience [17]. It has also shown that patient self-assessments closely match the standardized assessments performed by psychiatrists [18]. Therefore, adopting the EMA method to assess early response would be useful.

To ensure the reliability and validity of the data obtained through the EMA, it is crucial to employ self-reporting forms that have been previously validated. One such instrument is the Patient Health Questionnaire-4 (PHQ-4), which has demonstrated robust psychometric properties across diverse populations and settings [19]. The PHQ-4 is superior to other measures in its simplicity and practicality, making it an applicable choice in frequent, real-time assessments within the EMA framework.

In this study, we developed the Esketamine Continuing Assessment for Relapse Prevention (EsCARe), an EMA-based daily assessment application, which included the PHQ-4, and applied it in patients with TRD. By validating the baseline and day 1 treatment effects of ESK known from previous studies with the endpoints in EsCARe, we provide preliminary results on the validity of EsCARe and explore whether the early day 1 response to ESK found in patients with TRD can be confirmed using self-monitoring. This study hypothesizes that daily monitoring via EsCARe will reveal significant improvements in depressive symptoms post-treatment, providing a new dimension of understanding regarding the efficacy of ESK.

## 2. Results

### 2.1. Baseline Characteristics of Participants

From 20 May 2022 to 6 July 2023, 29 patients were eligible for the study. The sociodemographic and clinical characteristics of the 29 treated patients at baseline are shown in Table 1. We included 19 female and ten male patients with TRD (mean age 31.41 ± 13.69 years). The mean number of admissions due to MDD was 1.03 ± 2.06, and the mean PHQ-9 score was 19.69 ± 4.79, indicating severe depression [20]. Additionally, these patients experienced a significant burden associated with their condition, as seen by the prolonged duration of depression (mean ± standard deviation: 4.52 ± 3.94 years). Most of the patients (55.2%) suffered from other comorbid psychiatric conditions.

From the start of the current depressive episode, all patients were prescribed at least two antidepressants from two different classes. The mean number of concurrent antidepressants at the time of esketamine use was 2.21 ± 0.77, with five patients (17.2%) using a mood stabilizer as an augmentation (lithium: 4, lamotrigine: 1) and 22 patients (75.5%) using one or more atypical antipsychotics (quetiapine, aripiprazole, or olanzapine). All patients were using three or more psychiatric medications concurrently (including benzodiazepine, Z-drug for relieving anxiety and insomnia).

### 2.2. Esketamine Treatment

The starting dose of esketamine was 56 mg in all participants. The dose of esketamine was increased from 56 mg to 84 mg in four (13.8%) patients at a median of 8.0 (range, 8.0–15.0) days after initiation. At week 2, treatment was discontinued in one patient due to the withdrawal of consent. By week 4, a total of four patients (13.8%) had discontinued treatment, with two withdrawing consent, one presenting with suicidal ideation, and one dying by suicide.

Of the 24 patients who completed the induction period, six dropped out due to an insufficient treatment response, leaving 18 of 29 patients to start the optimization period. During the maintenance period, one patient died by suicide in week 20, and the remaining 17 patients completed all the treatments by week 24.

### 2.3. Clinical Outcome of Esketamine during the Induction Period

Table 2 presents the results of the repeated measured ANCOVA with age and sex as the covariates. Except for ISI, a significant time effect was found for depression, as measured by the PHQ-9 and the HAMD, and for anxiety, as measured by the GAD.

**Figure 1:**
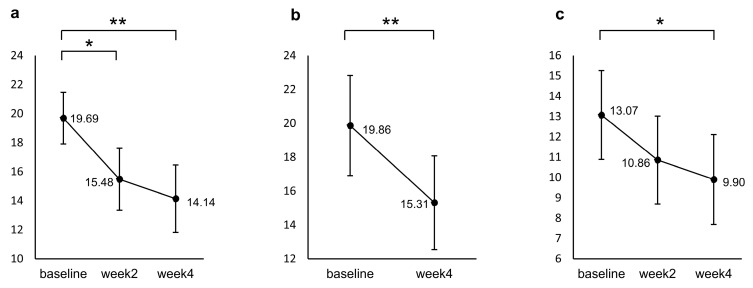
Changes from baseline in (**a**) PHQ-9, (**b**) HAMD, and (**c**) GAD during the induction period. The values shown are adjusted for the covariates (age and sex), and error bars indicate 95% confidence intervals. * *p* < 0.05, ** *p* < 0.001 (n = 29, the missing data were imputed using the last-observation-carried-forward approach).

Figure 1 shows the results of a post hoc test for the repeated measured ANCOVA. Participants’ PHQ-9 scores significantly decreased from baseline to week 2 (*p* = 0.001) and week 4 (*p* < 0.001), but no significant difference was found between week 2 and week 4 (*p* = 0.800). For the GAD, a significant decrease from baseline was identified only at week 4 (*p* = 0.009). The HAMD decreased significantly from baseline to week 4 (*p* < 0.001).

### 2.4. Safety and Tolerability of Esketamine

During the induction period, 23 patients reported 111 medically confirmed AEs, of which 98 (88.3%) were non-serious in 16 patients and 13 (11.7%) were severe in seven patients. The AEs occurring in patients were increased blood pressure or hypertension (three patients), anxiety (four patients), oversedation (one patient), dizziness (one patient), headache (two patients), nausea (three patients), and dissociation (one patient). All the AEs associated with intranasal esketamine administration were transient, self-limiting, and occurred on the administration day.

There was one suicide (death) and one self-harm with suicidal ideation during the induction period, both of which were determined to be due to the exacerbation of pre-existing symptoms. No cases of esketamine exposure during pregnancy or breastfeeding and no cases of esketamine abuse or dependence were observed. Three patients contracted COVID-19 while receiving esketamine and recovered with no other sequelae. No new safety signals were compared with the Phase 3 clinical trial data for esketamine [6,21,22].

### 2.5. Reliability and Validity of EsCARe Measurements

#### 2.5.1. Adherence to EsCARe

The mean number of data entries for days 3, 14, and 28 were 2.69 ± 0.60, 10.62 ± 3.31, and 19.41 ± 7.08, and the percentages of data entries were 89.6% (n = 78), 75.9% (n = 308), and 69.3% (n = 563), respectively. For the data completeness of each entry, we investigated whether all the required data were entered for each entry. We found that all the entries required data to be entered except for six entries (one during the first 3 days, two between 4 and 14 days, and three between 15 and 28 days).

#### 2.5.2. Distribution and Reliability of the Modified PHQ-4 and Sleep Diary in EsCARe

To investigate the reliability of the newly modified PHQ-4 and the 2-item sleep diary in the daily assessment module, we analyzed the distributions and descriptive statistics (mean, standard deviation, median, skewness, and kurtosis) of the PHQ-4 (modified) and the sleep diary (Table 3). The inputs utilized in the analysis were those performed on the day closest to each administration for the first week (two administrations). Considering their normal distribution, the skewness and kurtosis values of the PHQ-4 and the sleep diary from the daily assessment module of EsCARe were within the acceptable range. The modified PHQ-4 reliability using Cronbach’s α was good, and the Spearman’s test–retest correlation presented 0.87 (*p* < 0.001). The sleep diary, which consisted of two questions, had a Cronbach’s α of 0.37 and was not internally reliable, so we analyzed the scores for each item separately.

#### 2.5.3. Convergent and Concurrent Validity of EsCARe

As anticipated, Table 4 shows the significant positive correlations of the modified PHQ-4 in EsCARe with the HAMD (*p* = 0.001) and PHQ-9 (*p* < 0.001). Moreover, the correlation between the PHQ-4 and anxiety measured using the GAD was positive and statistically significant (*p* < 0.001).

In addition, of the two items in the sleep diary, only the results for sleep quality were significantly correlated with the ISI (*p* < 0.001), while the results for daytime drowsiness were not significantly correlated with either the ISI (*p* = 0.09) or the sleep quality scores (*p* = 0.268), as shown in Table 5.

### 2.6. Early Effectiveness of Esketamine by EsCARe

**Figure 2:**
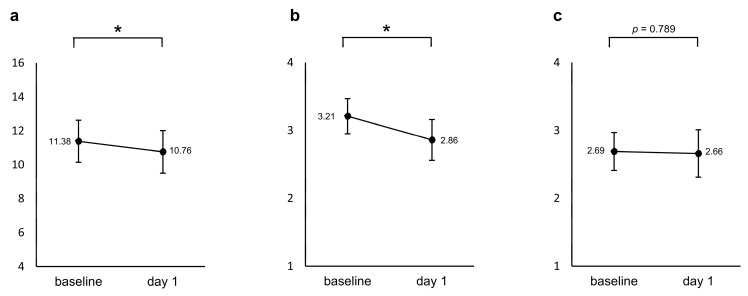
Changes from baseline in the (**a**) modified PHQ-4 and two items in the sleep diary ((**b**) sleep quality and (**c**) drowsiness) between baseline and the day after the first administration. The values shown are adjusted for the covariates (age and sex), and error bars indicate 95% confidence intervals. * *p* < 0.05.

A repeated measured ANCOVA with age and sex as covariates showed a significant decrease in the modified PHQ-4 from baseline to the day after the first administration (*F* = 4.265, *p* = 0.049, Partial η^2^ = 0.141). The sleep diary reported a significant decrease, i.e., an improvement in sleep quality (*F* = 5.813, *p* = 0.023, Partial η^2^ = 0.183), whereas no significant change was found for drowsiness (*F* = 0.073, *p* = 0.789, Partial η^2^ = 0.003), as shown in Figure 2.

## 3. Materials and Methods

### 3.1. Study Setting

Five sites in the Republic of Korea (Korea University Guro Hospital, Korea University Anam Hospital, Korea University Ansan Hospital, Kangbuk Samsung Hospital, and Hallym University Sacred Heart Hospital) recruited patients with TRD. All participating hospitals were tertiary care hospitals in the Republic of Korea that treat high-acuity patients referred from primary and secondary care hospitals and, therefore, have experience in treating many patients with TRD. Independent review boards of all the participating hospitals approved the study protocol and its amendments (Supplementary Table S1). The study was conducted based on the ethical principles of the Declaration of Helsinki. All the participants provided written informed consent before participation.

### 3.2. Study Design and Participants

This multi-center, open-label, single-arm, interventional study was conducted from 30 May 2022 to 6 July 2023. This study was designed to consider the real-world clinical environment in which EsCARe is applied, and the eligibility criteria were designed to meet the licensing conditions rather than the strict criteria considered in randomized controlled trials (RCTs). The study was conducted in adults aged 18–65 years.

The patients included in the study were those diagnosed with TRD, defined as an inadequate response to at least two different oral antidepressants of adequate dose and duration. The diagnosis of major depressive disorder (MDD) was verified through a comprehensive psychiatric assessment conducted by a board-certified psychiatrist, utilizing the criteria outlined in the Fifth Edition of the Diagnostic and Statistical Manual of Mental Disorders (DSM-5) [23].

#### 3.2.1. Inclusion Criteria

1. Participants were required to be prescribed esketamine and demonstrate the ability to self-administer the intranasal medication.
2. All the participants provided informed consent after fully understanding the study’s purpose and procedures.
3. Psychiatric comorbidities, such as generalized anxiety disorder (GAD), post-traumatic stress disorder (PTSD), and mild substance use disorder (excluding moderate-to-severe substance use disorder), were permitted as long as they did not interfere with the primary diagnosis of TRD or the safety of esketamine administration.
4. Concomitant medications, including mood stabilizers, antipsychotics, and sedative-hypnotics, were allowed if they had been part of the participant’s stable regimen for at least 4 weeks before the study began.

#### 3.2.2. Exclusion Criteria

5. Patients with a history of MDD with psychotic features, bipolar affective disorder, personality disorders, obsessive–compulsive disorder, intellectual disability, autism spectrum disorder, and moderate-to-severe substance use disorder diagnosed within the previous 6 months were excluded.
6. Any patient with a history of ketamine use disorder was excluded due to the potential for substance misuse.
7. Patients with medical conditions that could pose significant risks due to the potential side effects of esketamine, particularly those associated with elevated blood pressure or intracranial pressure, were excluded. This included individuals with aneurysmal vascular diseases, cerebrovascular disease with a history of stroke or transient ischemic attack, and coronary artery disease with recent significant cardiac events.
8. Participants whose depressive symptoms had previously demonstrated a nonresponse to esketamine therapy or electroconvulsive therapy (ECT) were excluded to ensure that the study focused on evaluating esketamine in a population that had not yet been fully assessed for this treatment.
9. Pregnant women were excluded due to the unknown risks of esketamine in pregnancy.

All the patients in the study could participate in the 6-month treatment period as recommended for ESK use. As the current study focused on the early effectiveness of esketamine, only the effects during the induction period were analyzed.

### 3.3. Study Procedures

Eligible patients received ESK according to the prescribing information approved by the Ministry of Food and Drug Safety in 2020. The administration of ESK was accompanied by the concomitant use of medications for treating depression, including pre-existing antidepressants, as permitted by the safety permit. These treatments were administered in a fully hospitalized or outpatient clinic setting.

A treatment session with ESK consisted of the self-administered nasal administration of this medication under the supervision of a healthcare professional and a post-administration observation. ESK was prescribed and administered at the dose required (up to 84 mg as esketamine hydrochloride) in each treatment session. All other administrations of ESK and the management of adverse events (AEs) were performed following the approved instructions for use in South Korea (“Recommended Dose” in Supplementary Materials). Before each administration of ESK, all the participants measured their baseline blood pressure to assess their medical stability. Following each dose, a healthcare professional monitored the patients for 120 min. These blood pressure measurements were repeated immediately after dosing, and at 40 min and 120 min post dose, and a cardiologist consultation was scheduled if elevated blood pressure (systolic ≥160 mmHg and/or diastolic ≥100 mmHg) persisted. The initial dose of esketamine was set at 56 mg. It was tailored to each individual throughout the treatment according to the following guidelines: during the induction phase (weeks 1–4) at 56 or 84 mg twice a week; during the optimization phase (weeks 5–8) at 56 or 84 mg once a week; and during the maintenance phase (weeks 9–24) at 56 or 84 mg every one or two weeks.

At the end of the induction phase, the decision to continue treatment was determined by evaluating its therapeutic benefits. The therapeutic benefits were judged by a ≥50% reduction compared with the total PHQ-9 measured at baseline. The dose was adjusted according to the effectiveness and tolerability of the previous dose. Patients were allowed to remain on the drug for up to 24 weeks and were evaluated at a follow-up visit within four weeks of treatment discontinuation. Specific dosages and uses are included in the Supplementary Material.

### 3.4. Assessments

#### 3.4.1. Esketamine Continuing Assessment for Relapse Prevention (EsCARe)

**Figure 3:**
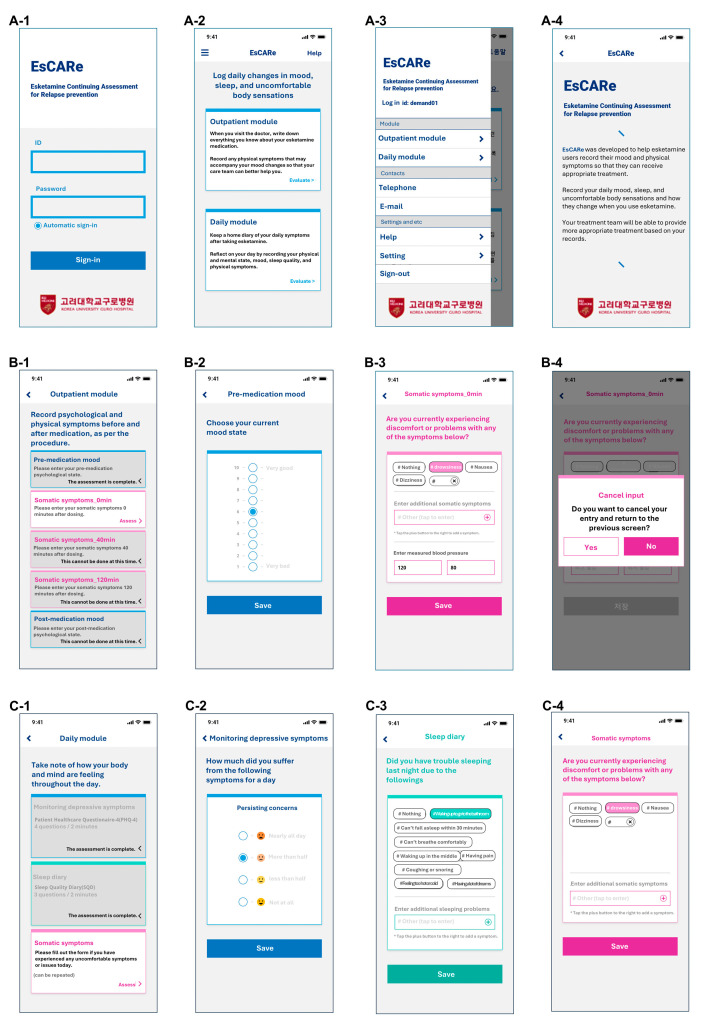
Screenshots of the EsCARe interface. (**A**) General system: (**A-1**) Login page of the EsCARe application; (**A-2**) Module selection page; (**A-3**) Menu from sidebar; (**A-4**) Description of EsCARe for participants. (**B**) Outpatient module: (**B-1**) Menu selection page; (**B-2**) Monitoring of depressive symptoms; (**B-3**) Monitoring of somatic symptoms; (**B-4**) Screen to cancel and re-input. (**C**) Daily module: (**C-1**) Menu selection page; (**C-2**) Monitoring of depressive symptoms; (**C-3**) Sleep diary; (**C-4**) Monitoring of somatic symptoms.

The EsCARe mobile-based strategy for evaluating the effectiveness and safety of esketamine, EsCARe, was initially designed by Junhyung Kim and Changsu Han. EsCARe was designed to be a simple daily monitoring assessment of depression and sleep by enabling participants to provide subjective reports within a brief timeframe efficiently. EsCARe (Demand, Gyeonggi-do, South Korea) was developed in 2022 and is available on Android and iOS. The general user interface of the EsCARe is shown in the screenshot in Figure 3.

EsCARe consists of two modules: the outpatient assessment module and the daily assessment module. The outpatient module supports the management of ESK. It consists of a component to monitor somatic symptoms and record blood pressure and AEs at the time of administration, 40 min after administration, and 2 h after administration. The monitoring somatic symptoms component is configured to support pre-dose blood pressure measurements as required by the ESK product license, as well as 40-min and 2-h post-dose monitoring, and to support the monitoring of AEs during monitoring. The monitoring of depressive symptoms component evaluates depressive symptoms on a 10-point scale (1 = very bad, 10 = very good) before administration and 2 h after administration. The patient’s completion of the outpatient assessment module is designed according to the ESK administration schedule and checked by the investigator.

The daily assessment module is a monitoring module that asks patients to report their mood, sleep, and physical symptoms daily. The daily assessment module consists of the monitoring of depressive symptoms component, a modified version of the PHQ-4 for screening depressive symptoms, the sleep diary component to assess sleep with two 4-point Likert scales concerning sleep quality, and one item concerning the factors disturbing sleep. Traditional PHQ-4 questions focus on a 2-week recall period, which can introduce recall bias and diminish the sensitivity to short-term changes. By adjusting the timeframe to daily reporting, the modified PHQ-4 aligns with the EMA methodology’s goal of capturing dynamic, real-time data. This approach allows for a more accurate and nuanced understanding of the early effects of esketamine treatment. In addition, monitoring of somatic symptoms is a component that assesses physical symptoms using self-reported items.

The monitoring of depressive symptoms component and the sleep diary component are designed to be input by the participant by setting the alarm to sound daily at the designated time (set by the participant) (Figure 3(C-1)). In the case of the monitoring of somatic symptoms component, participants can input data at any time they experience a physical symptom. A description of each module of EsCARe is presented in Table 6.

#### 3.4.2. Clinical Assessments

Clinical assessments were performed based on a predefined schedule (Table 7). The assessment tools were selected because they can be used in real-world clinical settings and have been used to validate efficacy in previous RCT studies [3,4,5]. In addition to the daily and outpatient assessment modules of EsCARe, clinical assessments were performed during hospital visits. The PHQ-9 for depressive symptoms, the 7-item Generalized Anxiety Disorder scale (GAD-7) for anxiety accompanying depression [24], and the Insomnia Severity Index (ISI) to assess insomnia were obtained at baseline and every 2 weeks during the induction period for a total of three times (baseline, week 2, and week 4) [25]. For the objective assessment, the Hamilton Depression Rating Scale (HAMD) was administered at baseline and week 4 by a trained rater [26]. Side effects and tolerability were assessed after each esketamine administration using a 4-item positive symptom subscale of the Brief Psychiatric Rating Scale (BPRS) [27] and the Modified Observer’s Assessment of Alertness/Sedation (MOAAS) scale [28].

### 3.5. Statistical Analyses

The descriptive statistics for participants’ baseline characteristics used means and standard deviations for the continuous variables and frequency distributions (number and proportion of patients in each category) for the categorical variables. To evaluate the reliability and validity of EsCARe, we performed analyses related to the items for depression and sleep in the daily assessment module of the in-app measurement of EsCARe, measured on the first and second administration days during the first week of the study, which is the main focus of the analysis. To assess adherence to EsCARe, the data obtained from the daily assessment module of EsCARe were examined for the frequency and completeness of data entry in the first week related to the first administration. For a further reliability and validity analysis, the missing data in the daily assessment module were imputed using the last-observation-carried-forward approach. The distributions of the in-app measurements for depression (modified PHQ-4) and sleep of EsCARe were presented with corresponding item skewness and kurtosis values for the first and second administration days during the first week. To assess the reliability of EsCARe, the internal reliability of the values measured on the first and second administration days during the first week was evaluated using Cronbach’s α. The test–retest reliability was analyzed using correlations between the values measured on the first and second administration days during the first week using the Spearman’s rank correlation analysis. Concerning the convergent validity, we analyzed the correlations among the in-app measurements in the daily assessment module of EsCARe concerning depression (modified PHQ-4) and sleep at the first administration day, HAMD, PHQ-9, GAD, ISI, and SDS at baseline using the Spearman’s rank correlation analysis. Concerning the clinical outcomes based on conventional assessments, changes in quantitative outcome measures from baseline to week 2 after the initiation of esketamine were examined using a repeated measures analysis of covariance (ANCOVA) for the PHQ-9, SDS, GAD, and ISI, controlling for age and sex. For the HAMD, comparisons were made between baseline and week 4 utilizing a repeated measures ANCOVA controlling for age and sex. Missing data were imputed using the last-observation-carried-forward approach. For the early effectiveness of esketamine, the depressive symptom monitoring items from the daily assessment module of EsCARe and the variables from the sleep diary were analyzed using a repeated measures ANCOVA, controlling for age and sex as the input variables on the first administration day and the following days. All the statistical analyses were performed using a commercial software package (IBM SPSS Statistics v28.0, AMOS v.28.0, for Windows, IBM Corporation, Armonk, NY, USA). Statistical significance was set at *p*-value < 0.05.

## 4. Discussion

This study aimed to evaluate the early effectiveness of esketamine via patient self-reports using EsCARe, and our findings indicated a significant reduction in depression and anxiety symptoms as early as the day following the first administration. Furthermore, we provided evidence that EsCARe, developed to assess early effectiveness, has sufficient reliability and validity for assessing depression and sleep. In addition, the results showed that both the modified PHQ-4 score for depression and the sleep diary score for sleep were significantly reduced from baseline in EsCARe on the day after ESK use.

A total of 29 patients participated in the current study; the duration of their current major depressive episode was more than 4 years, with an average of one hospitalization during this time. Clinical assessments at baseline showed scores of severe depressive symptoms on both the PHQ-9 and the HAMD for depression [20,29], moderate anxiety on the GAD [30], and moderate insomnia on the ISI [31]. The severity of depressive symptoms and comorbid anxiety and insomnia, combined with a higher chronicity, more suicide attempts, and reduced response–remission rates to antidepressants during a depressive episode, is well characterized by TRD [32]. In addition, more than half of the patients had a comorbidity of an anxiety disorder, and 75.5% of patients were on two or more antidepressants plus a mood stabilizer, such as lithium or atypical antipsychotics. These clinical features suggest that the study sample had significant treatment resistance to antidepressants [33].

The symptoms of depression significantly decreased after ESK administration at weeks 2 and 4, based on self-ratings. There was a numerical decrease in depression outcomes, albeit not statistically significant, between weeks 2 and 4, after Bonferroni correction, suggesting that the effect of ESK was more pronounced in the first 2 weeks. These results are similar to those of an RCT on the rapid reduction of symptoms of depression, which showed a significant difference from placebo in the first 11 days of treatment and no significant difference at 25 days of treatment [8]. Significant reductions in the HAMD, a clinician-rated measure, were also seen at week 4, and significant reductions in the GAD scores related to anxiety symptoms were also seen at week 4. These improvements in depressive and anxiety symptoms after esketamine use are consistent with several RCTs and real-world data that have previously shown benefits at 4 weeks for depression or anxiety [5,6,34,35].

On the other hand, no significant results were found in the ISI for sleep. Although not significant, the study also observed a trend toward a decrease in insomnia severity. Given the multiple positive effects of esketamine on sleep, improved sleep architecture [36], and reduced sleep latency [37]. Improved sleep quality in patients with major depressive disorders [38], the sample sizes of these previous studies were considerably larger than the current study, which may have affected the ability of this study to produce significant results.

For the adherence information for EsCARe, 89.6% of entries were entered for the first 3 days. In weeks 2 and 4, the proportion of entered entries was 75.9% and 69.3%, respectively. Based on previous studies of ecological momentary assessments, a data entry rate of over 60% is acceptable and supports the feasibility of utilizing early data to assess early effectiveness [39]. The distribution, skewness, and kurtosis values of both the modified PHQ-4 and the sleep diary in the daily assessment module of EsCARe were within the range of the standard for the normal distribution [40], which also supports the feasibility of applying EsCARe in patients with TRD using ESK.

Our research results showed that the modified PHQ-4 of EsCARe showed a good internal consistency based on Cronbach’s α (0.85), with values similar to those obtained in the English (Cronbach’s α ranging from 0.78 to 0.82) [19], Greek (Cronbach’s α = 0.80) [41], and Korean versions (Cronbach’s α = 0.79) of the original form of the PHQ-4 [42]. The modified PHQ-4 is challenging to compare to the original form because the timeframe was changed from 1 week to 1 day. However, this similarity can be used to support the internal reliability of the modified PHQ-4 because the proportion of the content in the questionnaire and answers is the same. The modified PHQ-4 also showed acceptable Pearson correlation coefficients (0.87) [43], with values similar to those obtained in the Korean version of the original PHQ-4 (0.83) [42].

Moreover, the convergent validity of the modified PHQ-4 showed significance supported by high Pearson correlation coefficients with existing depression (HAMD: 0.57, PHQ-9: 0.64) and anxiety scales (GAD: 0.67). This result seems relatively small compared with the correlation of the Korean PHQ-4 to the HAMD (0.76) [42]; given that the HAMD was assessed over the previous week and the PHQ-9 and GAD were assessed over the previous 2 weeks, we compared the modified PHQ-4 results on the last day of the period covered by each instrument. Previous studies of the Work and Social Adjustment Scale have reported different magnitudes of correlations with depression or obsession symptoms depending on the length of the assessment period [44]. Furthermore, given the high variability in assessment values that a short assessment period can introduce [45], the short assessment period of the modified PHQ-4 was a significant contributor to the relatively small correlations. Given that existing weekly assessments are not well suited to assessing the day-to-day effectiveness, which is what this study sought to investigate, and given that there is no instrument with confirmed validity at the day-to-day level, the results of this study provide minimal evidence that the modified PHQ-4 can be used to assess early effectiveness.

Concerning the sleep diary, the correlation between baseline and day 1 was significant, but Cronbach’s α of 0.35 was not acceptable. This may be because the item had only two items and consisted of questions about different domains: sleep quality and drowsiness [46]. Sleep quality and drowsiness have been suggested as different constructs in a previous polysomnography study [47]. The results of the Pearson’s correlation with the analyses also support our inference to assess the convergent validity of the two items separately; sleep quality was significantly correlated with ISI, whereas drowsiness was not. Six of the seven items of the ISI are related to sleep quality. In contrast, only one item is related to drowsiness; even then, it is covered by the construct of daily functioning [25]. Therefore, based on the low internal reliability of the sleep diary and the fact that only sleep quality was significantly correlated with the ISI, it is reasonable to analyze only the sleep quality item of the sleep diary.

A repeated measured ANCOVA of assessments at baseline and the day after the first dose of EsCARe, a previously validated measure of the early effectiveness of ESK, was significant for the modified PHQ-4 and sleep quality on the sleep diary but not for drowsiness on the sleep diary. The decrease in the modified PHQ-4 shows the possibility that the early effectiveness of the depressive symptoms identified in previous RCTs is significant not only on objective assessments but also on subjective assessments. Therefore, the early improvement assessed in these subjective assessments indicates the clinical utility of the ESK.

The early effectiveness of the ESK on sleep was investigated for the first time in this study. The improvement in subjective assessments of sleep quality in this study the day after dosing suggests the potential for new clinical utility. It has important implications for the use of the drug. Notably, the significant results for sleep quality the day after dosing, despite the lack of significant results on the Insomnia Severity Index (ISI), may indicate that ESK has rapid but potentially short-lived effects on sleep that are not fully captured by the ISI, which assesses longer-term sleep patterns. However, further studies are needed to confirm this, as the analysis was conducted only during part of the period.

Our study has limitations that require caution in interpreting the results. First, the design of this study was unblinded to a single arm. The unblinded design may have introduced bias, as patients were aware of the treatment being administered, possibly affecting their self-reported symptom severity. Second, the absence of a control group and the lack of randomization do not allow us to explain whether our findings relate to real-world clinical efficacy. Third, there is a potential for an increased Type I error due to multiple analyses on the same dataset. Fourth, the potential for non-random dropouts, likely influenced by the participants’ depression status, may have biased the results, affecting the accuracy of the study’s conclusions. Fifth, the small sample size cautions against interpreting the reliability or validity of the EsCARe we developed. Sixth, this study presents results based on subjective assessments. Future research should involve an RCT comparing daily mobile-based assessments with traditional weekly clinician assessments to validate our findings and explore the impact of assessment frequency on patient outcomes.

This study consistently presented the significant clinical benefit of previously reported measures of depression and anxiety associated with ESK in patients with TRD. It also showed the reliability and validity of EsCARe as a helpful tool in monitoring early treatment outcomes. Furthermore, EsCARe was effective in assessing the immediate effects of esketamine in patients with severe TRD, with improvements in depressive symptoms and sleep quality seen as early as day 1. These findings suggest the reliability and potential of EsCARe as a helpful tool in monitoring early treatment outcomes. EsCARe can improve patient care by providing real-time data, especially in real-world clinical settings where timely intervention is critical. This mobile-based assessment platform is expected to reduce the need for frequent clinic visits and help clinicians to make more informed treatment decisions, ultimately improving the management and outcomes of TRD in various healthcare settings.

## Figures and Tables

**Table 1:** Sociodemographic and clinical characteristics.

Variables (Continuous)	Mean	SD
Age (years)	31.41	13.69
Education (years)	13.76	1.90
Current major depressive episode duration (years)	4.52	3.94
Psychiatric admission (n)	1.03	2.06
Baseline clinical assessments		
PHQ-9	19.69	4.79
HAMD, 17 item	19.76	7.60
GAD	13.07	5.62
ISI	17.14	6.77
Variables (ordinal)	N	%
Female	19	65.5
Marital status		
Single	21	72.4
Married	6	20.7
Divorced/widowed	2	6.9
Occupation		
Unemployed	9	31.0
Employed	7	24.2
Student	13	44.8
Psychiatric comorbidities (duplicate possible)		
No	13	44.8
Anxiety disorders	16	55.2
Obsessive–compulsive and related disorders	1	3.5
Trauma-and stressor-related disorders	1	3.5
Feeding and eating disorders	2	6.9
History of psychiatric hospitalization (n)	11	37.9
Suicidality		
No	4	13.8
Ideation (responded to baseline HAMD item 3 with 1–3)	24	82.3
Attempts (responded to baseline HAMD item 3 with 4)	1	3.5
Concomitant drug at baseline (duplicate possible)		
Antidepressants		
SSRIs	21	72.4
SNRIs	24	82.8
Others	15	51.7
Other psychopharmacotherapy		
Mood stabilizers/anticonvulsants	4	13.8
Antipsychotics	22	75.9
Sedative hypnotics/anxiolytics	29	100

Notes: Hamilton Depression Rating Scale (HAMD), 9-item Patient Health Questionnaire (PHQ-9), 7-item Generalized Anxiety Disorder scale (GAD), Insomnia Severity Index (ISI), Selective serotonin reuptake inhibitor (SSRI), Serotonin norepinephrine reuptake inhibitor (SNRI).

**Table 2:** Results of the repeated measured ANCOVA in the clinical outcome of esketamine. (N = 29, the missing data were imputed using the last-observation-carried-forward approach, age and sex were included as covariates).

Variables	*F*	Effect Size (Partial η^2^)	*p*-Value	Significance of Covariates		Post-Hoc
				Age	Sex	
PHQ-9	12.2	0.49	<0.001	0.03	0.78	see Figure 1a
HAMD	17.03	0.40	<0.001	0.44	0.41	see Figure 1b
GAD	5.28	0.30	0.012	0.07	0.95	see Figure 1c
ISI	3.07	0.20	0.064	0.07	0.10	not significant

Notes: Analysis of Covariance (ANCOVA), Hamilton Depression Rating Scale (HAMD), 9-item Patient Health Questionnaire (PHQ-9), 7-item Generalized Anxiety Disorder scale (GAD), Insomnia Severity Index (ISI).

**Table 3:** Distribution and descriptive statistics and reliability of the modified PHQ-4 and sleep diary in EsCARe.

Variables	Mean	SD	Median	SK	ku	Cronbach’s α	Test–Retest Reliability
PHQ-4							
baseline	11.38	3.21	11	−0.51	−0.03	0.85	0.87 **
day 1	10.76	3.23	11	−0.11	−0.93	0.87	
Sleep diary							
baseline	5.91	1.08	6	0.22	−0.69	0.35	0.59 **
day 1	5.48	1.32	5	0.57	−0.62	0.37	

Notes. Modified 4-item Patient Health Questionnaire (PHQ-4), Standard deviation (SD), skewness (SK), kurtosis (ku). ** *p* < 0.001.

**Table 4:** Correlation between the modified PHQ-4 and other measures concerning depression and anxiety (n = 29).

Variable	PHQ-4	HAMD	PHQ-9	GAD
PHQ-4	-			
HAMD	0.56 *	-		
PHQ-9	0.64 **	0.47 *	-	
GAD	0.67 **	0.59 *	0.46 *	-

Notes: Hamilton Depression Rating Scale (HAMD), 9-item Patient Health Questionnaire (PHQ-9), 7-item Generalized Anxiety Disorder Scale (GAD). * *p* < 0.05, ** *p* < 0.001.

**Table 5:** Correlation between the items of the sleep dairy and the ISI (n = 29).

Variable	Sleep Quality	Drowsiness	ISI
Sleep Quality	-		
Drowsiness	0.21	-	
ISI	0.71 **	0.32	-

Notes: Insomnia severity scale (ISI), ** *p* < 0.001.

**Table 6:** Modules and contents of each component in EsCARe.

Module	Contents
Outpatient assessment	Monitoring of depressive symptoms
	- Before and after (at discharge) esketamine administration - 10-point Likert scale 1. Please indicate your current feeling (1 = very bad, 10 = very good)
	Monitoring of somatic symptoms
	- Somatic symptoms and blood pressure levels after esketamine administration (0 min, 40 min, and 2 h). Do you experience discomfort or problems with the symptoms below? #dissociation #drowsiness #nausea #dizziness #others (please specify) 2. Enter your current blood pressure reading
Daily assessment	Monitoring of depressive symptoms
	- Patient Healthcare Questionnaire—4 (used for daily assessment, partial amendment) - 4-point Likert scale, “How much did you suffer from the following symptoms for a day?” 1. Anxiety/agitation/2. Persisting concerns/3. Loss of interest/4. Feeling depressed and hopeless (1 = not at all, 2 = less than half the day, 3 = more than half the day, 4 = nearly all day)
	Sleep diary
	- 4-point Likert scale 1. How did you sleep last night? (1 = very good, 2 = good, 3 = bad, 4 = very bad) 2. How often did you feel drowsy while driving, eating, or socializing for a day? (1 = not at all, 2 = less than half, 3 = more than half, 4 = nearly all day) - Check the conditions you experienced (this is for monitoring insomnia-related patterns; multiple answers are possible) 3. Did you have trouble sleeping last night due to the following? #Can’t fall asleep within 30 min #Waking up in the middle of the night #Waking up to go to the bathroom #Can’t breathe comfortably #Having pain #Coughing or snoring #Feeling too hot or cold #Having a lot of dreams
	Monitory of somatic symptoms
	- Participants can input at any time every day (repeated input possible) 1. Do you experience discomfort or problems with the symptoms below? #dissociation #drowsiness #nausea #dizziness #others (please specify)

**Table 7:** Schedule for assessments.

	Every Visit	Daily	Baseline	Week 2	Week 4
EsCARe—Outpatient assessment module	✓				
EsCARe—Daily assessment module		✓			
9-item Patient Health Questionnaire			✓	✓	✓
7-item Generalized Anxiety Disorder scale			✓	✓	✓
Insomnia Severity Index			✓	✓	✓
Hamilton depression rating scale			✓		✓
4-item positive symptom subscale of the Brief Psychiatric Rating Scale	✓				
Modified Observer’s Assessment of Alertness/Sedation scale	✓				

## Data Availability

The data presented in this study are available on request from the corresponding author due to privacy.

## Supplementary Materials

The following supporting information can be downloaded at: https://www.mdpi.com/article/10.3390/ph17091143/s1, Table S1: List of participating hospitals and approvals from each independent review board; Recommend Dose: Approved instructions for esketamine nasal spray in South Korea.
