# Supplementary material for: A Multi-Center, Open-Label, Single-Arm Study to Investigate the Early Effectiveness of Esketamine Nasal Spray in Patients with Treatment-Resistant Depression Using a Mobile Self-Monitoring Application
Source: Pharmaceuticals (Basel). 2024 Aug 30;17(9):1143. doi: 10.3390/ph17091143 (PMC11434815; doi:10.3390/ph17091143)
Supplement: Supplementary file 1 [file pharmaceuticals-17-01143-s001.zip › pharmaceuticals-3149895-supplementary.pdf]

Supplementary Materials

Supplementary Table S1. List of participating hospitals and approvals from each independent review board

Name of Hospitals	IRB Number
Korea University Guro Hospital	2021GR0413
Korea University Anam Hospital	2021AN0551
Korea University Ansan hospital	2021AS0354
Kangbuk Samsung Hospital (Sungkyunkwan University School of Medicine)	KBSMC 2022-03-015
Hallym University Sacred Heart Hospital	2021-09-020-002

Recommended Dose

- The drug is administered together with an oral antidepressant. The treatment process consists of an induction phase (weeks 1 to 4) and maintenance phase (weeks 5 to up to 24). The recommended dose of this drug during the induction phase (weeks 1 to 4) for the treatment of TRD are shown in the table below.

Day	Dose	Instructions for Dose Titration
Day 1	56 mg	
Day 4	56 or 84 mg	As determined by the investigator based on efficacy and tolerability, the dose may be increased to 84 mg or maintained at 56 mg.
Day 8	56 or 84 mg	As determined by the investigator based on efficacy and tolerability, the dose may be increased to 84 mg (if the dose of Day 4 is 56 mg) or maintained at the dose of Day 4 or decreased to 56 mg (if the dose of Day 4 is 84 mg).
Day 11	56 or 84 mg	As determined by the investigator based on efficacy and tolerability, the dose may be increased to 84 mg (if the dose of Day 8 is 56 mg) or maintained at the dose of Day 8 or decreased to 56 mg (if the dose of Day 8 is 84 mg).
Day 15	56 or 84 mg	If required due to tolerability, a dose decrease from 84 mg to 56 mg is allowed. If the dose of Day 11 is 56 mg, a dose increase is not allowed on Day 15.
Day 18, 22, 25	56 or 84 mg	After Day 15, a dose increase from 56 mg is not allowed. If required due to tolerance, dose down-titration from 84 mg to 56 mg from Day 15 until Day 25 is additionally allowed only once.

- At the end of the induction phase, whether to continue treatment is determined by evaluating its therapeutic benefits. Therapeutic benefits are judged by a ≥50% reduction compared to the total score of the 9-item Patient Health Questionnaire (PHQ-9) measured at baseline. The dose is adjusted according to the efficacy and tolerability of the previous dose. The frequency of intranasal treatment sessions for the first four weeks of the maintenance phase (weeks 5 to 8) is reduced from twice a week to once a week. Dose increase is not allowed after week 8 during the maintenance phase. If required due to tolerability, a dose decrease from the previous dose to 28 mg is allowed from week 9 until the phase ends.

- The frequency of intranasal treatment sessions after week 9 is adjusted at fixed four-week intervals (starting at week 8 and thereafter at week 12, 16, and 20) based on the guidelines below (if applicable).

1) Week 8:
    A. If the total score of PHQ-9 is ≤7,
    - Change the frequency of esketamine treatment sessions to once every two weeks (i.e. the next intranasal treatment session after week 8 is at week 10)

    B. If the total score of PHQ-9 is >7,
    - Do not change the frequency of esketamine treatment sessions

    C. If PHQ-9 assessment is missed at week 8, use the total score of PHQ-9 obtained before week 8 to determine if the frequency of treatment sessions at week 8 needs to be changed.
    - If the total score of PHQ-9 is ≤7, reduce the frequency of intranasal treatment sessions to once every two weeks for the next four weeks (i.e. the next treatment sessions are at week 10 and week 12).
    - If the total score of PHQ-9 is >7, maintain the frequency of intranasal treatment sessions to once a week and no further change in the frequency of treatment sessions is allowed for the next four weeks.

2) From week 12 to follow-up visits at four-week intervals:
    A. If the total score of PHQ-9 is ≤7,
    - Change the frequency of esketamine treatment sessions to once every two weeks if the frequency was once a week.

    B. If the total score of PHQ-9 is >7,
    - Do not change the frequency of esketamine treatment sessions if the frequency was once a week.
    - Change the frequency of esketamine treatment sessions to once a week if the frequency was once every two weeks.

    C. If PHQ-9 assessment is missed at the week, use the total score of PHQ-9 obtained before the week to determine if the frequency of treatment sessions at week 8 needs to be changed.
    - If the total score of PHQ-9 is ≤7,
    : Change the frequency of esketamine treatment sessions to once every two weeks if the frequency was once a week.
    : Do not change the frequency of esketamine treatment sessions if the frequency was once every two weeks.
    - If the total score of PHQ-9 is >7,
    : Do not change the frequency of esketamine treatment sessions if the frequency was once a week.
    : Change the frequency of esketamine treatment sessions to once a week if the frequency was once every two weeks.

- During the maintenance phase, a change in the frequency of treatment sessions from once a week to once every two weeks is allowed up to three times. Thereafter, if the treatment given once every two weeks is not effective for the participant, the frequency of treatment sessions will be maintained at once a week for the remaining period of the study.

The drug is administered for each dose as described in the table below:

Intranasal Treatment	Administration Time [a]		
	0[a]	5 min.	10 min.
Intranasal Device[b]	First	Second	Third
Esketamine 56mg	Spray esketamine once into each nasal cavity	Spray esketamine once into each nasal cavity	No device required
Esketamine 84mg	Spray esketamine once into each nasal cavity	Spray esketamine once into each nasal cavity	Spray esketamine once into each nasal cavity

a. Time 0 is defined as the time when first spraying the drug into one nasal cavity using the first intranasal device.

b. Use a single device at each time. Each intranasal device contains the volume for two sprays. An individual intranasal device contains 28 mg of esketamine for two sprays:14 mg for each spray.

- If a participant misses >4 consecutive intranasal administration sessions and/or ≥4 consecutive PHQ-9 assessments during the optimization/maintenance phase, the study for the participant is discontinued and an early withdrawal visit is completed, then the follow-up phase begins. If the participant misses the scheduled visit (hospital visit or telephone call) during the optimization/maintenance phase without informing the reason, a staff member will try to contact the participant to ascertain if the participant is still willing to participate in the study and if so, schedule the next visit. During the phone conversation, information regarding adverse reactions and concomitant medicines will be collected.
